# Modelling, Analysis, and Optimization of the Effects of Pulsed Electrophoretic Deposition Parameters on TiO_2_ Films Properties Using Desirability Optimization Methodology

**DOI:** 10.3390/ma13225160

**Published:** 2020-11-16

**Authors:** Nesrine Barbana, Adel Ben Youssef, Mohamed Ali Rezgui, Latifa Bousselmi, Mohammad Al-Addous

**Affiliations:** 1 Department of Environmental Process Engineering, Institute of Environmental Technology, Technische Universität Berlin, Secr. KF 2, Straße des 17. Juni 135, 10623 Berlin, Germany; 2 Higher National Engineering School of Tunis, University of Tunis, Tunis 1008, Tunisia; adel.benyoussef@esstt.rnu.tn (A.B.Y.); mohamedali.rezgui@gmail.com (M.A.R.); 3 Wastewaters and Environment Laboratory, Center for Water Research and Technologies, Technopark of Borj-Cedria, Soliman 8020, Tunisia; latifa.bousselmi.certe@gmail.com; 4 Department of Energy Engineering, School of Natural Resources Engineering and Management, German-Jordanian University, Amman 11180, Jordan; mohammad.addous@gju.edu.jo

**Keywords:** pulsed electrophoretic deposition, photocatalysis, scratch test, conversion layer, RSM, desirability optimization methodology

## Abstract

Titanium dioxide thin films immobilized over treated stainless steel were prepared using the pulsed electrophoretic deposition technique. The effects of process parameters (deposition time, applied voltage, initial concentration, and duty cycle) on photocatalytic efficiency and adhesion properties were investigated. To optimize the multiple properties of the thin film, a response surface methodology was combined with a desirability optimization methodology. Additionally, a quadratic model was established based on response surface analysis. The precision of the models was defined based on the analysis of variance (ANOVA), R^2^, and the normal plot of residuals. Then, a desirability function was used to optimize the multiple responses of the TiO_2_ thin film. The optimum values of applied voltage, catalyst concentration, duty cycle, and deposition time were 4 V, 16.34 g/L, 90% *DC*, and 150 s, respectively. Under these conditions, the decolorization efficiency of tested dye solution reached 82.75%. The values of critical charges *L*_C1_, *L*_C2,_ and *L*_C3_ were 5.9 N, 12.5 N, and 16.7 N, respectively.

## 1. Introduction

Stainless steel (SS-316L) has been used extensively in a variety of applications in industries along with everyday life, due to its low corrosion rate and excellent mechanical properties [1]. It has been employed as a substrate for TiO_2_ films due to its electrical properties [2,3,4].

In recent decades, various coating methods have been employed to deposit titanium dioxides such as sputtering [5], spin coating [6], dip coating [7], anodization [8,9,10], chemical vapor deposition (CVD) [11], plasma-enhanced CVD (PECVD) [12], atomic layer deposition (ALD) [13], physical vapor deposition (PVD) [14], and pulsed laser deposition [15]. The Electrophoretic Deposition (EPD) technique, well-matched with varied applications, has recently attracted great attention to process innovative materials such as ceramics, metals, living cells, biological materials, and polymers [16,17] because of its low cost, high productivity, short processing time, basic equipment, and simple set up. During the EPD process, the charged particles in the suspension are moved under a direct current electric field to be deposited over the oppositely charged electrode [18]. Up to now, few studies have considered EPD of TiO_2_ by organic suspensions [19,20] or mixtures of organic solvents and water [21,22]. An aqueous medium can cause the formation of bubbles in the deposit due to the electrochemical reactions at the surface of the electrodes. To overcome this problem, pulsed voltage could be used instead of direct voltage. Besra et al. [23] have confirmed based on their study on aqueous EPD of alumina suspension that using pulsed direct current as an alternative of continuous direct current is a suitable and effective approach to control and suppress the bubble formation in the deposit. At proper pulse widths and/or duty cycle, bubble-free deposits were attained. Naturally, adhesion between TiO_2_ and bare SS is poor due to incompatibility between these two [24,25]. To overcome this adhesion problem, the surface needs to be etched chemically. Among the conventional applied techniques is the creation of a conversion layer (CL) before the coating [26].

In this present work, pulsed EPD of TiO_2_ coating on an SS substrate with a CL has been studied. Pulsed EPD is an interesting process to deposit a controlled coating by adjustment of the deposition parameters. Limited works have been prepared with the aim to see the effect of EPD factors on the photocatalytic degradation of pollutants [27,28]. The impact of these parameters on decolorization efficiency and adhesion of the film must be studied to find the optimal conditions for the deposition of the TiO_2_ layer.

The majority of researchers have defined their experimental results according to the influence of one parameter at a time [29]. This research aims to evaluate the effects of pulsed EPD parameters: applied voltage, deposition time, initial concentration, and duty cycle.

Among the methodologies to achieve the optimum results is the Response Surface Methodology (RSM) [28,30,31]. An experimental design methodology must be economical to extract the maximum amount of complex information, a significant reduction in experimental time, thus saving material as well as personnel cost. This is the first reported study dealing with the production of TiO_2_ nanoparticles and the optimization of pulsed EPD parameters to accomplish maximum decolorization efficiency and critical charge values based on a set of pulsed EPD parameters.

In a previous experiment [26], the conversion layer has led to improving the film adhesion and resulted in a degradation percentage of 60%. The key objective of this work is to develop a practical model that predicts maximum decolorization efficiency and critical charge values based on a set of pulsed EPD parameters. The optimization method was based on RSM followed by a desirability optimization methodology as a practical technique when optimizing multiple responses [32,33]. The current technique is an interesting process to deposit a low-cost active photocatalyst that showed no adhesion difficulty.

## 2. Materials and Methods

### 2.1. Substrate Preparation

A raw sheet of stainless steel (316L) was cut into discs of 3 mm thickness and 15 mm diameter. Substrates were first polished by 400, 600, 800, and 1200 sandpaper grits and cleaned using deionized water. Then, they were dried in the air. The result was a smooth surface. Afterward, the substrates were ultrasonically cleaned in acetone for 10 min. Next, they were immersed in a conversion bath [26] for 35 min at 60 to 70 °C to produce a passive layer. The conversion bath is composed of 5 mL of sulfuric acid (H_2_SO_4_) mixed with 0.126 g of hydrated sodium thiosulfate (Na_2_S_2_O_3_·5H_2_O) and 0.6 mL of propargylic alcohol (C_3_H_4_O) in a 100 mL volumetric flask. Then, the samples were rinsed in demineralized water and dried at 120 °C for 60 min. To stabilize the coating, the samples were heat-treated at 450 °C for 2 h [26]. The conversion layer was used to simultaneously increase the adhesion of the TiO_2_ film and the efficiency of the photocatalytic treatment.

### 2.2. Pulsed Electrophoretic Deposition

**Figure 1:**
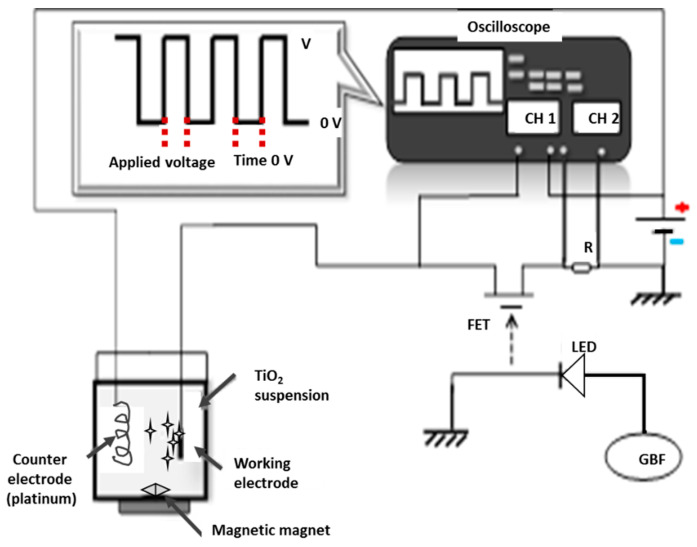
Schematic illustration of the pulse circuit generator and working electrophoresis cell [26].

Pulsed electrophoretic deposition (pulsed EPD) was performed to depose the TiO_2_ layer onto the prepared substrate. The setup is described in Figure 1. The counter electrode was made of platinum. The cathode was a SS substrate. The two electrodes were 20 mm apart in a 40 mL glass beaker. The electrolyte was prepared by mixing the TiO_2_ powder with 40 mL of deionized water. The powder was prepared by controlled hydrolysis of TiCl_4_ followed by dialysis through a cellulose membrane [26]. The mixture was stirred vigorously for 30 min before being used to deposit TiO_2_ thin films over pre-functioned stainless steel (316L) with a conversion layer (Fe_2_O_3_). The deposition was performed at ambient temperature (25 °C). Then, the prepared electrodes were dried in ambient air for 24 h. 

### 2.3. Characterization of the Film

Scratch tests were carried out for the characterization of adhesion. They were performed at room temperature via the Nanoindenter NHT^2^ (CSM Instruments, Peseux, Switzerland) using a Rockwell spherical diamond tip with a radius of 50 µm. The tests were achieved by progressive load from 30 mN to 15 N. The loading speed was 30 N/min over a distance of 3 mm.

Based on the loads at which cracking and delamination occurred, in addition to the variation in the frictional force, the adhesive force of the coating is determined. The results are confirmed by the optical observations of the scratch track [34,35].

The different adhesion failures can be identified by three critical charges:

✓ *L*_C1_ is the load at which the first cracks occurred (cohesive failure).
✓ *L*_C2_ is the load at which the film starts to delaminate at the edge level of the scratch track (adhesion failure) [36].
✓ *L*_C3_ [28] occur when the damage of the film exceeds 50% [37].

### 2.4. Degradation Experiments 

In each experiment, a solution of textile dye: amido black-10B (C_22_H_14_N_6_Na_2_O_9_S_2_; M = 616.5; AB-10B with a concentration of 10 mg/L was used to assess the photocatalytic activity at room temperature. The adsorption equilibrium of AB-10B on the catalysts was reached during 30 min of immersion of the electrode in the dark. Shortly thereafter, the solution was exposed to a UV lamp (HPK 125W, Cathodeon, Cambridge, UK) with *λ* = 365.5 nm. A noticeable peak at 617 nm and other small peaks at 226 and 318 nm characterize the absorbance spectra of AB-10B. The concentration of the AB-10B was monitored using a UV-Vis spectrophotometer by examining the main absorbance at 617 nm. The decolorization efficiency (*De*) was calculated as follows (Equation (1)):

(1) $$De\;\left(\%\right)=\left(\frac{Abs_{0}-Abs_{t}}{Abs_{0}}\right)\times 100$$

where *Abs_0_* and *Abs_t_* point to the solution absorption at 617 nm at UV irradiation time 0 and t, correspondingly.

### 2.5. Experimental Design

The multivariate experimental design provides appropriate information about the suitable operating conditions with minimum experimental work. The Central Composite Design (CCD) [38] is a commonly used form of RSM that was employed to investigate the effects of independent variables on the 4 responses (see below).

Four factors were selected as operating parameters in the study. They are the initial TiO_2_ concentration, the deposition time, the duty cycle, and the applied voltage referred to as A, B, C, and D, respectively.

The duty cycle is defined by the following equation (Equation (2)):

(2) $$DC\;=\frac{T_{ON}}{T_{ON}+T_{OFF}}$$

where $T_{ON}$ is the portion of the cycle for which the voltage is ON and $T_{OFF}$ is the portion of the cycle for which the voltage is null.

By varying $T_{ON}$ and $T_{OFF}$ we managed to adjust the frequency of pulse application at a constant voltage.

The experimental range and levels of these independent variables are listed in Table 1. The experimental domain for each factor was chosen according to prior knowledge of our system [26], the results of other research [39], and the performance of the used photocatalytic reactor [40,41].

CCD includes three parts: a two-level full (2^*k*^, where *k* is the number of input factors) or fractional factorial, axial points, and center points [38]. In our case, we used a two-level full factorial design (2^4^ points for a four-factor design (*k* = 4)), 2^*k*−1^ axial points, and 6 center points. This design allows us to estimate the main effects of input factors and their interactions on output responses [42]. It was adopted to maximize four multiple responses which were the photocatalytic efficiency of the thin film (*De* (%)) and three critical charges *L*_C1_ (N), *L*_C2_ (N), and *L*_C3_ (N).

CCD can have diverse design properties by monitoring alpha “*α*”: the value of the distance from each axial point to the center of the design. The commonly selected value of *α* is *α* = *k*^0.5^ or *α* = (NF)^0.25^, with NF being the number of factorial points in a *k* factor design. In our case, *α* is equal to 2. The factorial points help to estimate the interaction terms. The center points deliver information about the existence of curvature in the method, with which the axial points enable us to estimate the quadratic terms.

Table 2 shows the completely experimental design and real responses of the experiments involved in this study. The 30 manipulations were prepared in random order. We are interested in maximizing all four responses.

The Minitab software (version 17.1.0) was applied to carry out the statistical analysis and create the model of the experimental data. The methodology adopted for the experimental modeling involved a sequential study of the experimental results. Initially, the analysis of the observations (*De*, *L*_C1_, *L*_C2,_ and *L*_C3_) was carried out at the end of a complete factorial design 2^4^. Then, the design was improved by 6 center points. Finally, in this study, we present the analysis obtained by a CCD design in which we added the axial points.

The standard error (S), the correlation coefficient (R^2^), and the adjusted correlation coefficient (R^2^adj) were used to determine the quality of developed models. They show how well the model fits the data. These values can help to choose the most suitable model. The obtained models were approved by ANOVA (analysis of variance), regression analysis, and normal plot of residuals. A *p*-value of less than 0.05 shows that model terms are significant.

The smaller the value, the more important its corresponding coefficient, and the bigger the influence on the response variable [43]. As a result, we can analyze the relationship between the predictor and the response. 

When a regression model fails to describe the functional relationship between the response variable and the experimental factors and if important model terms such as quadratic terms or interactions are not considered, we are talking about lack of fit. 

The Durbin–Watson statistic (DW-statistic) is used to test the existence of autocorrelation in residuals. It reports a test statistic “d” between 0 and 4. A test statistic equal to 2 signifies no autocorrelation. If it is from 0 to 2, it agrees with positive autocorrelation, and from 2 to 4, we are talking about negative autocorrelation.

The optimization process included evaluating the statistically designed combinations, fitting the experimental data to the predicted responses, calculating the response of the fitted model, and checking the suitability of the model.

## 3. Results and Discussion 

The experimental design was developed to evaluate the effect of the operating parameters (initial concentration, deposition time, *DC,* and applied voltage) on the decolorization efficiency (*De* %) of the TiO_2_ film and its three critical charges (*L*_C1_, *L*_C2,_ and *L*_C3_).

The statistical data analysis was split into two phases. First, we studied the effect and the interactions of different factors using ANOVA. Second, the results were optimized in such a way to maximize the four responses.

### 3.1. Analysis of Variance (ANOVA) and Response Surface Analysis

ANOVA analysis of reduced models of the four responses *De* (%), *L*_C1_, *L*_C2_, and *L*_C3_ based on a CCD experimental design is summarized in Table 3. A quadratic model was applied to provide an optimum combination of the pre-treatment parameters. 

The experimental design for De (%) in terms of the four independent variables was modeled by the polynomial equations (Equation (3)) with a probability of 5% (*p* < 0.05).

(3) $$\begin{array}{ll} De & =52.549\;+0.461\;A-5.715\;B-6.320\;C-9.084\;D-3.756\;A\times A\;+\;1.828\;C\times C\\ & -\;2.440\;D\times D-2.581\;A\times B\;-4.109\;B\times C\;+5.235\;B\times D\;+7.094\;C\times D \end{array}$$

The interactions (AC, AD, and B^2^) are removed from the reduced model because they are not relevant. Despite it being irrelevant, factor A was kept in the model for reasons of hierarchy.

The values of R^2^ and R^2^ (adj) were superior to 97%, which proves the existence of a strong correlation between the experimental results and the reduced model [44].

DW-statistic value of the reduced model was d = 1.76 > dU = 1.64 (*α* = 5%), thus indicating no serial correlation. The *p*-value of 0.08 indicated that no significant autocorrelation remained in the residuals.

**Figure 2:**
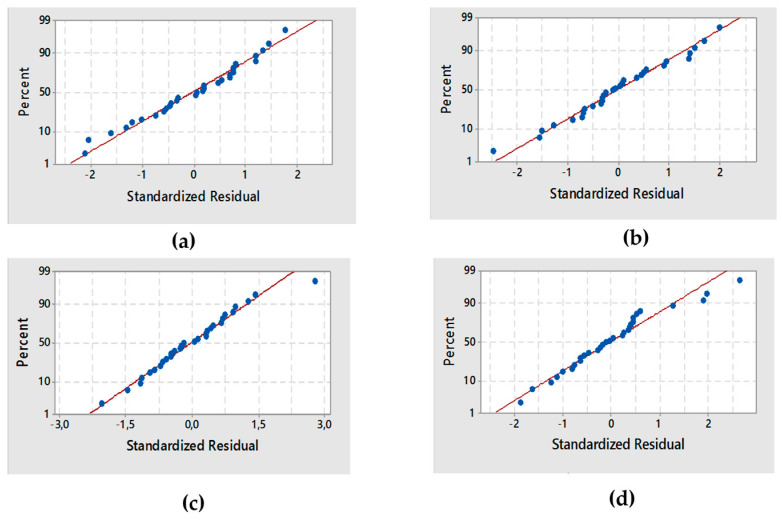
Normal probability plot for the four responses: (**a**) decolorization efficiency De, (**b**) L_C1_, (**c**) L_C2_, (**d**) L_C3_.

The residual analysis of the response surface design was determined based on the diagnostic plot (Figure 2a). 

The plot confirmed that the statistical assumptions fitted the analysis data. Given the normal probability of the residuals, we noticed that the standard deviations between the actual and the predicted response values followed a normal distribution.

The results from Figure 2a correspond to the normal distribution of principal errors. The residuals decreased near to a straight line, highlighting the conformity of the experimental results. 

The experimental design for *L*_C1_ in terms of the four independent variables was modeled by the polynomial equations (Equation (4)) with a probability of 5% (*p* < 0.05).

(4) $$\begin{array}{c} L_{C1}=3.6217-0.2771\;A-0.3596\;B-0.0479\;C-0.3879\;D-0.1647A\times A+\\ 0.3041\;B\times B\;-0.1397\;C\times C+\;0.0453\;D\times D-0.2119\;A\times B\;-0.0931\;A\times C\;+\\ 0.5669\;A\times D\;+0.1744\;B\times C\;-0.1106\;B\times D-\;0.1194\;C\;\times D \end{array}$$

The interactions (C, AC, and D^2^) were detached from the reduced model because they were not relevant.

The values of R^2^ and R^2^ (adj) were superior to 91%, thus attesting that there was a strong correlation between the experimental results and the reduced model.

The value of DW-statistic of the reduced model of *L*_C1_ was d = 2.15 > dU = 1.64 (*α* = 5%) demonstrating that there is no serial correlation. A *p*-value of 0.43 showed that there was no significant autocorrelation in the residuals.

The diagnostic plot illustrated in Figure 2b shows how the statistical assumptions fit the analysis data. The standard deviations between the actual and the predicted *L*_C1_ values led to a normal distribution. Moreover, the residuals were near to a straight line, thereby confirming that the experimental results were normal.

The experimental design for *L*_C2_, in terms of the four independent variables, was modeled by the polynomial equations (Equation (5)) with a probability of 5% (*p* < 0.05).

(5) $$\begin{array}{ll} L_{C2} & =6.562\;-0.1875\;A-\;0.5450\;B-0.1158\;C-1.0550\;D-0.3604\;A\times A\;\\ & +\;0.2808\;B\times B\;+\;0.0897\;C\times C+\;0.3533\;D\times D-0.128\;A\times B\\ & -\;0.332\;A\times C+0.628\;A\times D\;+0.120\;B\times C\;-\;0.207\;B\times D\\ & -\;0.522\;C\;\times D\; \end{array}$$

The interactions (AB, BC, BD, and C^2^) were removed from the reduced model because they were not relevant. Although factors A and C were irrelevant, they were saved in the model for causes of hierarchy.

The values of R^2^ and R^2^ (adj) were superior to 89%, thus demonstrating that a strong correlation was established between the experimental results and the reduced model.

The value of DW-statistic of the reduced model of *L*_C2_ was d = 2.56 > dU = 1.64 (*α* = 5%), which suggested no serial correlation. Based on the *p*-value of 0.9315, there was an absence of significant autocorrelation remaining in the residuals. The residual analysis of the response surface design of *L*_C2_ was determined from the plot illustrated in (Figure 2c). 

The standard deviations between the actual and the predicted *L*_C2_ values produced a normal distribution. Additionally, the residuals were reduced near to a straight line, attesting to the normality of experimental results. 

The experimental design for *L*_C3_ in terms of the four independent variables was modeled by the polynomial equation (Equation (6)) with a probability of 5% (*p* < 0.05).

(6) $$\begin{array}{ll} L_{C3} & =9.522+\;0.0962\;A+0.4554\;B+0.3213\;C-0.5321\;D-\;0.2580\;A\times A\\ & +\;0.0782\;B\times B\;+0.4232\;C\times C+\;0.9382\;D\times D+0.4531\;A\times B\\ & -\;0.1556\;A\times C-0.5906\;A\times D\;-0.5156\;B\times C\;-\;0.2881\;B\times D\\ & -\;0.0719\;C\times D \end{array}$$

The interactions (CD and B^2^) were detached from the reduced model due to irrelevance. Although it is not significant, factor A was presented in the model for causes of hierarchy. 

The values of R^2^and R^2^ (adj) were superior to 94%, thus displaying/attesting to the existence of a strong correlation between the experimental results and the reduced model.

The value of DW-statistic of the reduced model of *L*_C3_ was d = 2.15 > dU = 1.64 (*α* = 5%), thus suggesting no serial correlation. Additionally, the *p*-value of 0.6006 proved the absence of significant autocorrelation remaining in the residuals. 

The residual analysis of the response surface design of *L*_C3_ was determined from the plot illustrated in (Figure 2d). The points in the normal probability plots of the residuals were reasonably close to a straight line. This confirmed the normal distribution of the errors and the effect of the input variables on the responses. 

**Figure 3:**
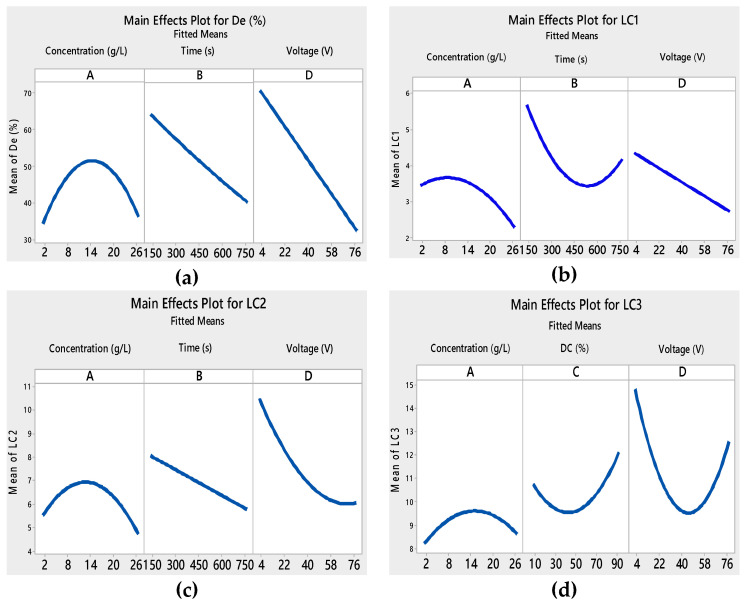
Main effects plots for different responses: (**a**) degradation, (**b**) *L*_C1_, (**c**) *L*_C2_, and (**d**) *L*_C3_.

In this study, the main effects plots for the fitted values of the four responses are presented in Figure 3. *DC* (%) did not appear in the main effects plots of *De* (%), *L*_C1,_ and *L*_C2_ responses because it presented no major effect. Each level of *DC* (%) from 10 to 90% affected the response in the same way, and the average response was the same for all factor levels. 

In the example of initial concentration, we noticed in Figure 3a that degradation efficiency constantly increased with the increase in the initial concentration of the suspension from 2 to 14 g L^−1^. This result may be linked to the structural and morphological properties of TiO_2_ films, which differ from one another. Increasing the TiO_2_ concentration produced a non-uniform and thick TiO_2_ film, thus forming more cracks [45].

The catalyst aggregation caused a smaller number of accessible active sites on the film surface, which explained the decrease in the *De* (%). The colorant degradation rate *De* (%) was influenced by the photocatalyst quantity in a photocatalytic process [46].

Increasing the initial concentration led to an increase in the amount of the deposited TiO_2_, and created agglomerates on the substrate’s surface. Particles are apt to agglomerate at higher concentrations [47].

We also noticed that the adhesion properties of the film became weaker (Figure 3b–d) when the initial concentration was above 14 g L^−1^. This was related to the cracks on the surface of the film.

In the case of the applied voltage, we found that in Figure 3a–c, the *De* (%), *L*_C1_, and *L*_C2_ decreased with the increase in voltage. When the applied voltage was above the water splitting potential, gas evolution was likely to occur at the electrodes. There was formation of H_2_-gas on the deposition cathode, which had damaging effects on the coatings. The gas bubbles inhibited good adhesion to the substrate and generated cracks in the deposit [48].

**Figure 4:**
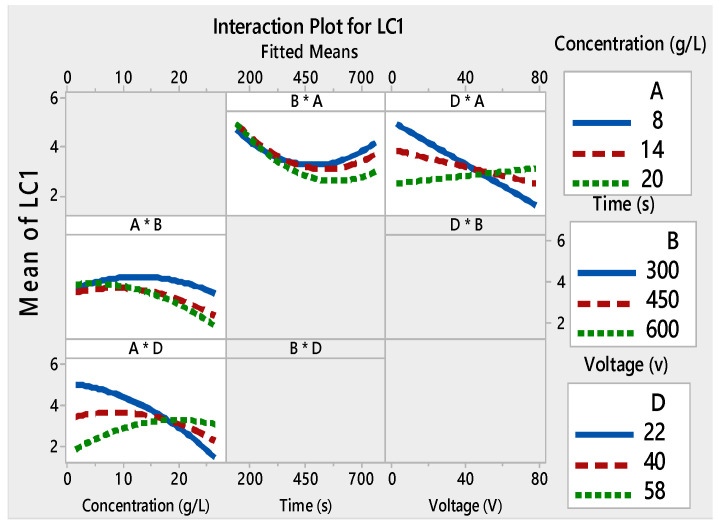
Interaction plots for L_C1_ ((*) refers to multiplication sign (×)).

**Figure 5:**
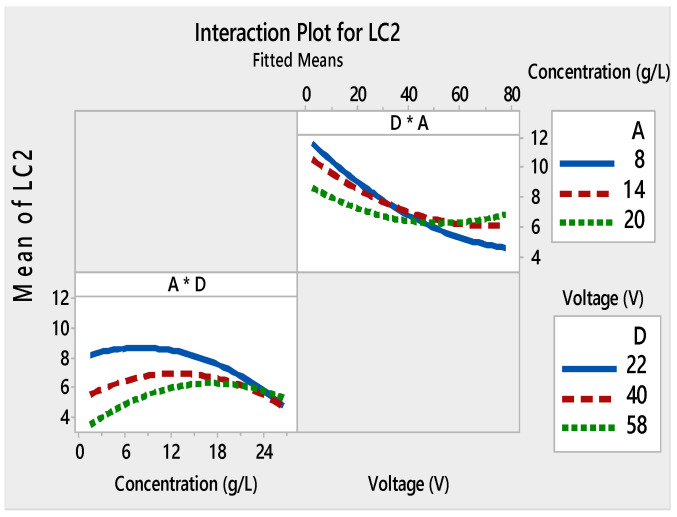
Interaction plots for L_C2_ ((*) refers to multiplication sign (×)).

**Figure 6:**
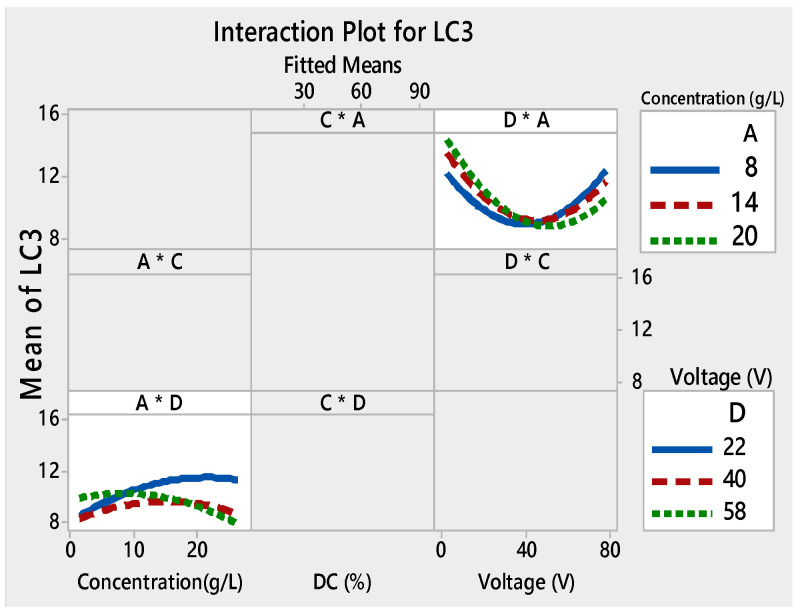
Interaction plots for L_C3_ ((*) refers to multiplication sign (×)).

**Figure 7:**
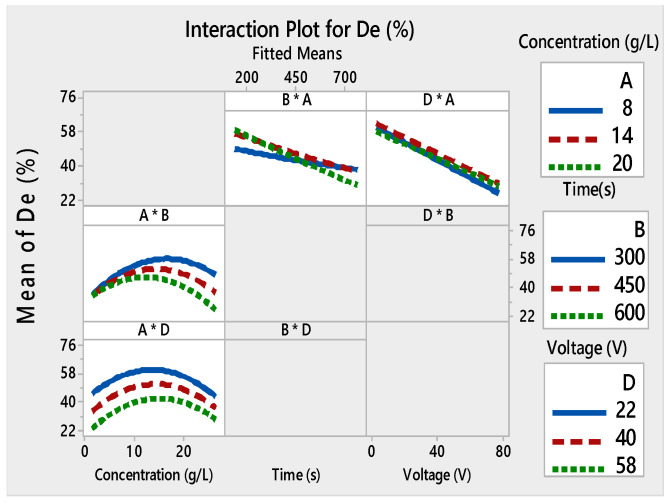
Interaction plots for De (%) ((*) refers to multiplication sign (×)).

Films with good adhesion and high decolorization efficiency were formed within very short deposition times as seen in Figure 3a–c. Depending on the voltage and the deposition time, the thickness of the film changed [49,50]. As a result, the adhesion of the TiO_2_ film to the substrate and the photocatalytic properties were modified. The interaction curves (Figure 4, Figure 5, Figure 6 and Figure 7) created a matrix of interaction graphs for the factors of each response. This interaction diagram was a diagram of averages for each factor level. However, the level of the second factor was kept constant. 

Furthermore, these figures show that *L*_C1_ and *L*_C2_ values increased at low voltages (4–40V), and initial concentrations varied from 8 to 14 g/L. However, starting from about 40V, this phenomenon was inverted. The small decrease in *L*_C_ with an increase in applied voltage can be attributed to several factors. To have a regular and homogeneous deposit, the particles must be dispersed. Higher voltage made more cracks on the TiO_2_ surface. This suggested that the film adhesion was poor. These mixtures between the TiO_2_ concentration and the applied voltages monitor the speed and the height of each pulse throughout the deposition process. 

Figure 6 illustrates the interaction plots of different factors on *L*_C3_. *DC* (%) was present as the main effect factor for *L*_C3_ (Figure 3), but it had no interaction with the other factors (Figure 6). This demonstrated that the main effects and interactions were independent of each other. Main effects can exist without interactions.

However, there was a slight interaction between the voltage and the concentration at a constant voltage, and between the time and the concentration at the constant time. At the low voltage and a concentration of 14 g/L, the *De* (%) was the highest (Figure 7).

There was no interaction between the applied voltage and the concentration (D × A) at constant concentrations. The parallel lines for the interaction plot of D × A (Figure 7) were high which indicated that the degree of interaction was also high.

The interaction plot for the four responses showed that there was a strong interaction between the initial concentration used for the deposition of the film and the two other relevant factors: time and voltage.

Given the link between the applied voltage (D) and the degradation efficiency (*De* (%)) of the films, it was clear that at high voltage (58 V), the *De* (%) was between 22% and 40%, yet it overstepped 50% at low voltage (22 V). The electrophoretic potential affected the nanocrystalline TiO_2_ film morphology [48]. The voltage and the initial concentration had the greatest impact on the efficiency of the film.

Electrophoretic deposition produces smooth and homogeneous films. Increasing the voltage yield further produces films that are too thick to produce enough adhesion and contact forces between particles. Consequently, cracks will be formed all over the surface of the film [51]. Additionally, raising the applied voltage reduces the creation of H atom due to a by-production, which cuts off the lattice of the Ti-O-Ti bond. As a result, there is a low quantity of TiO_2_ film deposition and a low *De* (%) [52].

In the case of TiO_2_ concentration, it was found that the *De* (%) continuously increased with the increase in TiO_2_ concentration from 8 to 20 g/L and also at a deposition time between 150 and 450 s. Nevertheless, we noticed a greater degradation when the deposition time was over 450 s and the concentration was as low as 8 to 14 g/L. This effect may be linked to the morphological properties of TiO_2_ films.

When the time of deposition increased, *De* (%) simply decreased. This negative effect may be a result of the relationship between deposition time and the deposited weight of TiO_2_ on the substrates.

### 3.2. Optimization and Verification of the Results

**Figure 8:**
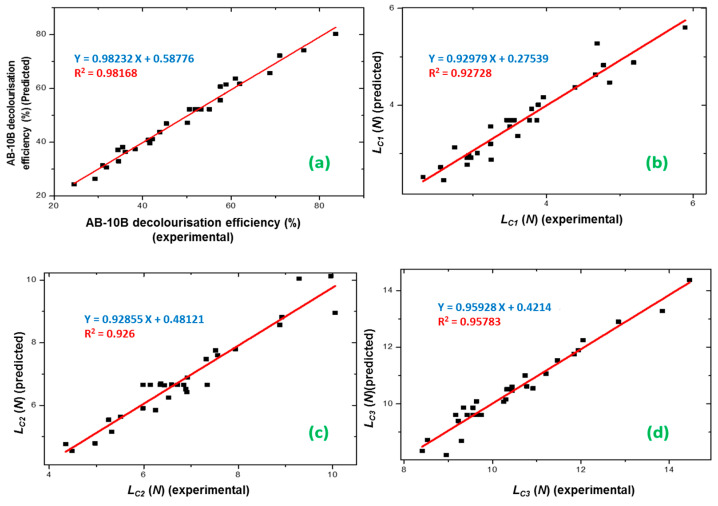
Graphical representations of the experimental values of (**a**) AB-10B decolorization efficiency (%), (**b**) L_C1_, (**c**) L_C2_, and (**d**) L_C3_ values plotted against the predicted values derived from the central composite design resulting equation.

The polynomial models (Equations (3)–(6)) obtained in this study were used for the experimental optimization of the process parameters (initial concentration, time, *DC,* and voltage) to maximize photocatalytic efficiency (*De* (%)) and the adhesion properties (*L*_C1_, *L*_C2_, and *L*_C3_) of the TiO_2_ film. The graphical representations of the experimental *De* (%), *L*_C1_, *L*_C2,_ and *L*_C3_ versus the predicted values are shown in Figure 8. 

There was a resemblance between the experimental data and the expected results in the four responses. The desirability method [53] was applied by changing each expected response, $Y_{i}$, into a function without units confined by 0 < $d_{i}$ < 1.

The d_i_ values for *L*_C1_, *L*_C2_, and *L*_C3_ were equal to 1, which gives more credibility to the responses. However, if $d_{i}$ = 0, this shows an undesired response [53]. Usually, the desirability plot characterizes the optimal region that fulfills the essential predetermined conditions.

The main objective of this work is the deposition of a TiO_2_ film on treated SS substrate under optimal process conditions to maximize decolorization efficiency and film adhesion to the substrate. The single desirability for each response $d_{i}$ was calculated using Equations (3) to (6). The optimization function in the Minitab software was applied to achieve optimal conditions. 

The vertical red lines in the graph represent the current factor settings as well as the displayed numbers on the top of the column. The horizontal blue lines and numbers indicate the responses for the current factor level.

The ultimate goal of this optimization was to achieve maximum values of different responses that altogether fulfilled all desirable conditions. This objective was reached based on two steps. First, obtaining the desirability for all the responses (*De*, *L*_C1_, *L*_C2,_ and *L*_C3_), then maximizing the desirability and optimal value identification.

**Figure 9:**
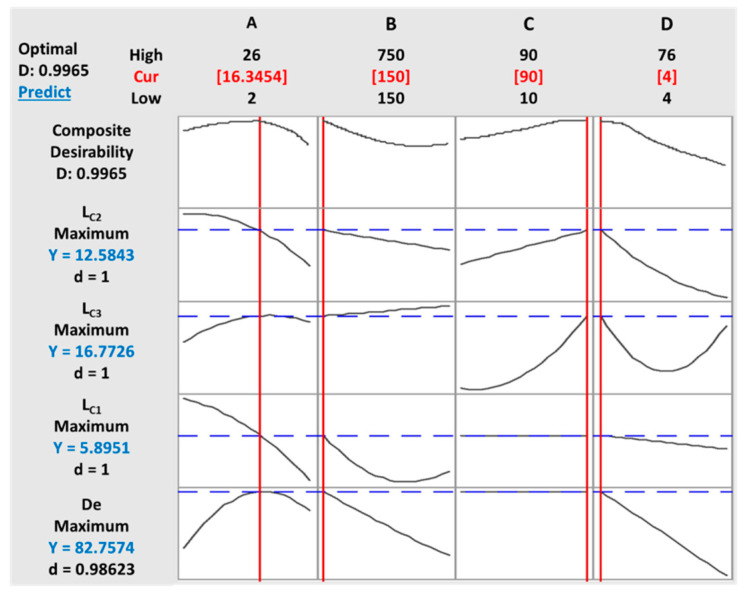
Optimization plot of multiple responses under the defined parameters: (**A**) initial concentration, (**B**) time, (**C**) duty cycle (DC), and (**D**) voltage.

Table 4 showed the input variables, their limits, and their goals settings. Different solutions can be attained in a desirability-based approach. The ones with high desirability are selected. Figure 9 shows the results of the desirability function approach.

The optimum levels under the defined parameters were found to be a concentration of 16.34 g/L of TiO_2_, a deposition time of 150 s, a duty cycle of 90%, and an applied voltage of 4 V. At optimum conditions, the model predicted an 82.75% decolorization efficiency of AB-10B. The lack-of-fit was greater than 0.05 in all four responses, implying that there was no difference between the experimental and predicted models.

The use of low voltage (4 V) was very common in the aqueous system [54]. However, water electrolysis might occur. No gas was developed on the surface of the electrode because the deposition time (150 s) was too small. If the process is well controlled, there will be an adequate formation of cracks on the surface of the deposited film. This will be justified by SEM images. The electrophoresis mobility was affected by the initial concentration [39]. Consequently, a concentration of 16 g/L was sufficient to have a uniform TiO_2_ film at low voltage and in a short deposition time.

At a low duty cycle, the *T_ON_* time was too short. The alternative relationship between *T_ON_* and *T_OFF_* considerably determined the development of the TiO_2_ film. Besra et al. [55] believed that the gas progress in the case of pulse voltage electrolysis was an active process where the gas generation on the electrode surface changed after each *T_OFF_* and may be different from previous and following ones. Such phenomena will produce separate micro- and nanosized bubbles homogeneously dispersed. 

The coalescence of small bubbles was energetically unfavorable, and their incorporation in the deposit produced no macro-bubbles. The 90% duty cycle was nearly collinear with the constant voltage because the long duty cycle approached a constant voltage deposition [56].

We reproduced the deposition of TiO_2_ film in optimum operation levels four times (Table 5) to approve the validity of the suggested models.

The average decolorization of AB-10B from the confirmation experiments was 80.075%, which agrees with the projected value of 82.75%. Therefore, the optimum point determined by RSM was effectively confirmed.

**Figure 10:**
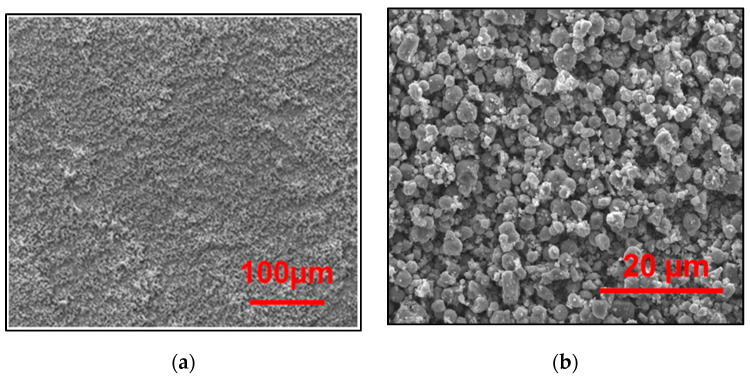
SEM images of the TiO_2_/ CL at optimum operating conditions: (**a**) Scale of 100 μm, and (**b**) Scale of 20 μm.

Figure 10a shows the SEM micrographs of the nanocrystalline TiO_2_ films deposited in optimization conditions. Decreasing the voltage further produced a film that was too thin. The adhesion and contact forces between particles were better. As a result, we obtained a smooth and crack-free film.

Too high electric-field intensity when applying a pulsed EPD can create an extremely high deposition rate such that the TiO_2_ particles will not be deposited at appropriate positions, resulting in poor thickness homogeneity.

Additionally, Figure 10b shows that TiO_2_ thin films consist of small aggregated particles, resulting in a highly porous volume. RSM helped to optimize the photocatalytic decolorization of AB-10B with TiO_2_ films and provide good adhesion properties of the prepared film using pulsed EPD.

## 4. Conclusions

The photocatalytic performance of TiO_2_ films has been greatly improved due to the microstructure of the surface. In this work, a novel supported photocatalyst was prepared by TiO_2_ deposition using pulsed EPD. The effects of different parameters (deposition time, applied voltage, initial concentration, and duty cycle) on the thin film properties were investigated. The statistical design was used for the multi-response optimization of TiO_2_ thin films. We tried to maximize the photocatalytic efficiency (*De* (%)) and the adhesion properties (*L*_C1_, *L*_C2,_ and *L*_C3_) of TiO_2_ films. An empirical relationship between the response and independent variables was established. The experimental design of the responses according to the four independent variables was modeled by polynomial equations with a probability of 5%. Both the correlation coefficient (R^2^) and the adjusted correlation coefficient (R^2^adj) values were good, which meant that the proposed quadratic models matched the experimental data very well.

The optimum levels were found to be a concentration of 16.34 g/L of TiO_2_, a deposition time of 150 s, a duty cycle of 90%, and an applied voltage of 4 V. The degradation efficiency reached 82.75% after maximum desired responses.

In this study, the projected multi-objective optimization approach proved to be a powerful methodology. Enhanced modeling steps help to obtain the best correlation. It offers to scientific researchers and industrials a supportive multi-objective optimization technique in case of various combinations of input.

## Figures and Tables

**Table 1:** Experimental range and levels of independent factors used in Central Composite Design (CCD).

Coded Variables	Factors	Units	Coded Level				
			−*α*	−1	0	+1	+*α*
A	Initial Concentration	g/L	2	8	14	20	26
B	Time	s	150	300	450	600	750
C	Duty Cycle (*DC*)	%	10	30	50	70	90
D	Voltage	V	4	22	40	58	76

**Table 2:** Experimental conditions and actual values of different responses in CCD design.

	Variables				Responses			
Stander Order	A	B	C	D	*De* (%)	*L*_C1_(N)	*L*_C2_(N)	*L*_C3_(N)
1	−1	−1	−1	−1	76.39	4.69	8.88	9.23
2	1	−1	−1	−1	83.57	3.89	7.56	9.56
3	−1	1	−1	−1	68.77	4.67	7.32	10.74
4	1	1	−1	−1	58.87	2.32	6.89	13.84
5	−1	−1	1	−1	57.58	4.78	9.29	11.21
6	1	−1	1	−1	61.96	3.24	7.52	11.47
7	−1	1	1	−1	31.82	5.19	10.05	10.78
8	1	1	1	−1	29.23	2.92	6.59	12.85
9	−1	−1	−1	1	30.96	3.61	6.53	9.64
10	1	−1	−1	1	38.36	4.39	7.94	8.96
11	−1	1	−1	1	43.9	2.56	5.32	10.92
12	1	1	−1	1	41.68	3.25	6.36	10.45
13	−1	−1	1	1	42.26	2.97	5.51	11.85
14	1	−1	1	1	50.09	3.8	6.25	9.35
15	−1	1	1	1	34.42	2.94	4.48	10.3
16	1	1	1	1	34.62	2.75	4.35	10.26
17	0	0	0	0	52.09	3.46	6.35	9.56
18	0	0	0	0	51.89	3.56	7.34	9.17
19	0	0	0	0	50.57	3.55	6.14	9.75
20	0	0	0	0	53.24	3.87	6.71	9.64
21	0	0	0	0	55.09	3.52	5.98	9.43
22	0	0	0	0	51.84	3.77	6.85	9.58
23	−*α*	0	0	0	36.13	3.5	5.26	8.41
24	+*α*	0	0	0	35.52	2.6	4.97	8.53
25	0	-*α*	0	0	60.96	5.89	8.92	9.3
26	0	+*α*	0	0	41.31	3.96	6.44	10.33
27	0	0	−*α*	0	70.95	3.24	6.92	10.45
28	0	0	+*α*	0	45.37	3.06	6.911	11.94
29	0	0	0	−*α*	57.62	4.86	9.96	14.46
30	0	0	0	+*α*	24.56	2.92	5.98	12.05

**Table 3:** Analysis of variance: reduced models of the four responses.

	De%				*L*_C1_				*L*_C2_				*L*_C3_			
Source of Variation	Sum of Squares	Df	Mean Square	*p*-Value	Sum of Squares	Df	Mean Square	*p*-Value	Sum of Squares	Df	Mean Square	*p*-Value	Sum of Squares	Df	Mean Square	*p*-Value
A	5.09682	1	5.09682	0.2039	1.8426	1	1.8426	0.0004	0.84375	1	0.84375	0.1282	0.222337	1	0.222337	0.0664
B	783.869	1	783.869	0.0000	3.1032	1	3.1032	0.0001	7.1286	1	7.1286	0.0032	4.9777	1	4.9777	0.0001
C	958.618	1	958.618	0.0000	0.0551042	1	0.0551042	0.2039	0.321554	1	0.321554	0.3119	2.47684	1	2.47684	0.0006
D	1980.53	1	1980.53	0.0000	3.6115	1	3.6115	0.0001	26.7126	1	26.7126	0.0002	6.7947	1	6.7947	0.0000
A × A	395.026	1	395.026	0.0001	0.820288	1	0.820288	0.0024	3.90084	1	3.90084	0.0112	2.02907	1	2.02907	0.0009
A × B	106.606	1	106.606	0.0011	0.718256	1	0.718256	0.0033					3.28516	1	3.28516	0.0003
A × C									1.7689	1	1.7689	0.0461	0.387506	1	0.387506	0.0272
A × D					5.14156	1	5.14156	0.0000	6.3001	1	6.3001	0.0042	5.58141	1	5.58141	0.0001
B × B					2.47966	1	2.47966	0.0002	2.01107	1	2.01107	0.0375				
B × C	270.109	1	270.109	0.0001	0.486506	1	0.486506	0.0074					4.25391	1	4.25391	0.0002
B × D	438.484	1	438.484	0.0000	0.195806	1	0.195806	0.0401					1.32826	1	1.32826	0.0023
C × C	93.5315	1	93.5315	0.0015	0.598163	1	0.598163	0.0048					4.75407	1	4.75407	0.0001
C × D	805.141	1	805.141	0.0000	0.228006	1	0.228006	0.0311	4.3681	1	4.3681	0.0090				
D × D	166.676	1	166.676	0.0004					3.24633	1	3.24633	0.0160	24.064	1	24.064	0.0000
Lack-of-fit	96.7267	13	7.44052	0.1085	0.912965	12	0.0760804	0.1206	3.17031	14	0.226451	0.6075	1.97794	12	0.164828	0.0663
Pure error	11.9433	5	2.38867		0.129083	5	0.0258167		1.27188	5	0.254377		0.203083	5	0.0406167	
Total	6144.9	29			20.8783	29			62.1753	29			63.0796	29		
R ^2^	98%				95%				92%				96%			
R ^2^ (adj)	97%				91%				89.09%				94%			
S	1.54				0.16				0.504358				0.201536			
Mean absolute error	1.58				0.15				0.297567				0.200067			
DW-statistic	1.76 (*p* = 0.08)				2.15 (*p* = 0.43)				2.56707 (*p* = 0.9315)				2.15913 (*p* = 0.6006)			
Lag 1 residual autocorrelation	0.09				−0.13				−0.297881				−0.0798249			

adj: adjusted

**Table 4:** Goals and parameter range for optimization of deposition levels.

Response	Goal	Lower	Target	Upper	Weight	Importance
*L*_C3_	Maximum	8.41	14.46	14.46	1	1
*L*_C2_	Maximum	4.35	10.05	10.05	1	1
*L*_C1_	Maximum	2.32	5.89	5.89	1	1
*De* (%)	Maximum	24.56	83.57	83.57	1	1

**Table 5:** Validation of the optimum conditions.

	Optimal Responses	Exp 1	Exp 2	Exp 3	Exp 4
*L*_C1_	5.9	5.2	4.8	5.3	5
*L*_C2_	12.5	10.5	11.3	12	11.8
*L*_C3_	16.7	14.3	14	14.8	14.5
*De* (%)	82.75	75.3	78	86	81
